# Medical Gossip

**Published:** 1870-04

**Authors:** 


### Medical Gossip.

Dr. Elsberg has devised a "pocket laryngoscope," which strikes us as excellent. It is beautifully simple, and must come into general use. He states, what we are prepared to indorse, that "every physician can learn with ten minutes' practice, to perform laryngoscopy in all ordinary cases." The instrument he recommends ought to be, and probably is, afforded at a figure very much below that asked for the cumbrous affairs now in use.——A foreign journal details a case of cancer cure by application of gastric juice produced by establishing a fistulous opening in the stomach of a dog. Time, about a month. Professor Schiff prefers for this purpose the pancreatic juice.——Compressed air is recommended as at once a sedative to circulation and respiration, and a tonic to the bronchial mucous membrane, in maladies of the respiratory passages. It is said to be an excitant of digestion also.——The Royal Danish Society of Science has offered among other prizes, one of $170 for the best essay on the movement of the air in a system of ventilation. It is open to the competition of all nations and nearly all tongues, but must be handed in before October next.——There were fifty-eight boiler explosions in England during 1869, by which eighty-six persons were killed and one hundred and twenty-six injured.——The Zircon light is to be used in the Pneumatic Tunnel under Broadway. A single light will illuminate an entire car with great brilliancy. Two small cylinders containing compressed oxygen and hydrogen are carried on each car, and from them proceed tubes causing the gases to impinge on a pencil of zircon one-quarter of an inch long and one-eighth of an inch in diameter. It burns without any readjustment, and a single pencil will last three months. This plan of illumination is worth looking at by those in search of light.——We have found among the most efficient diuretics, increasing both the solid and fluid excretion, the mucilage prepared from sliced leaves of the common prickly pear (*Cactus opuntia*). This plant is indigenous as far north as the banks of the Hudson river, and is readily cultivated in this section. It is entirely unirritating, and by the use of half a tumblerful of the mucilage several times a day, we have repeatedly increased the amount of urine from below the normal standard to eighty or ninety ounces in the twenty-four hours. No noxious results have at any time been observed in the course of a hundred or more trials. From careful observations we find that the total amount of solid matter excreted is considerably increased, although not by any means to the extent of the fluid. The remedy has, at all events, the advantage of being cheap and abundant — a circumstance not to be overlooked in these days, when the druggists get all the patient's money, and the doctors little but promises to pay hereafter.——Hypodermic injections of ergotine ($\frac{1}{2}$ to 3 grs.) have been used by Langenbeck in treatment of aneurism, and Dr. Drasche has arrested hæmoptysis instantaneously by the same device.——Workers in copper are said to be exempt from epidemic cholera. Where is *Judaeus Apella?*——M. Jeannel states the addition of $\frac{1}{5000}$th part of tartaric acid renders syrup of iodide of iron clear when it has decomposed, and at the same time notably diminishes the inky taste.——In a case of tetanus from wound of the fingers, Mr. Maunder cut across the median, radial and ulnar nerves in the lower fourth of the arm, instead of amputating.——The hydrate of chloral is now being used by everybody as an anodyne and soporific. Doses from five grains to a drachm. We have used it freely in large doses in numerous cases, but regret that we cannot go into raptures over it. At present writing, we much prefer a good article of chloric ether prepared in the old way, and not merely dilute chloroform, as the manner of most druggists is. The difference is greater than that between alcohol, whisky and brandy.——Speaking of chloroform, the Pharmaceutical Association at Washington, D.C., prepare an *Elixir Chloroform* thus:

℞ Chloroform, tinct. opi., tinct. camph., sp. ammon. ar., aa ℥ iss;
Oil of cinnamon, - - - - - - *m* xx;
Brandy (*sic*), - - - - - - - ℥ ij;

M.S.—Half a fluid drachm, more or less.

——One part of amber in one and a half parts of sulphide of carbon, is said to make an excellent cement. Apply the liquid with a brush, and press the surfaces together. It dries almost immediately.——The bisulphite of soda and carbolic acid are recommended in the treatment of trichiniasis. Nobody yet reports the use of chloral herein.——Prof. Wood, in his monograph on the hemp plant of North America, concludes it an excellent substitute for the foreign article, being both cheaply prepared and active. He uses the male seedling plants from Kentucky, and proved his faith in it by dangerous experiments upon himself. The active principle is a soft greenish resin, and is sufficiently active in quarter and grain doses.——Once more carbolic acid, $\frac{2}{3}$ grain in 20 minims of water, administered hypodermically, is said to cure intermittent.——In aphthæ, stomatitis, ottorhœa, etc., "A Country Physician" recommends in the *Bulletin:*

| | | |
|---|---|---|
| ℞ | Acid gallic, - - - - - - | ℈iss; |
| | Glycerin, - - - - - - | ℥j; |
| | Liq. iod. co., - - - - - - | ʒss; |
| | Aq. dest., - - - - - - | ℥j; |

M.S.—Apply with a camel's-hair brush several times daily.

——M. Vassin attributes five distinct varieties of cutaneous eruption to the continued use of the bromide of potassium.——M. Simon, of Hamburg, points out the occurrence of cerebral hæmorrhage and softening, after inhalation of the mixed gases arising from the combustion of coal.——Our learned correspondent Z. C. McElroy, M.D., writes to Prof. Potter (*Buffalo Medical Journal*, February) an explanation of the *modus operandi* of organic poison from the stand-point of his views of the physical basis of life.——A compromise of the questions involved between the line and staff officers of the navy is anticipated. The surgical position will be improved; it can scarcely be made worse.

THE Medical Department of the Howard University, D. C., (colored) held its commencement exercises March 3. This is the second year of the institution, and it numbers, in this department, some thirty students, white, mixed, black, and female. They have a substantial three-story brick building with French roof, occupied in common with the Freedman's hospital. The property which the University now owns, free from debt, is worth $1,000,000. Which reminds us that Senator Sumner, in his recent onslaught upon the Medical Society of the District of Columbia, urging the repeal of its charter, etc., committed, as it appears, a grave mistake. The "offences" he charges upon that society were really the doings of an entirely distinct voluntary "Association," which is not a chartered body.

It is to be regretted that the Massachusetts Senator would not retract his words, when the matter was fully explained to him. The officers of the Medical Society, however, have fully ventilated the subject in the Washington press.

One Dr. Donkin, of the University of Durham, (Eng.,) claims to have been the first to employ the milk diet for the relief and cure of diabetes. He must be pretty well along in years then, for we personally know of its use for this purpose more than quarter of a century ago, and even then it was spoken of as an old practice.

Inhalation of the aethereal tincture of valerian is recommended in hysteria.

Many of the journals are "recurring to first principles," vindicating calomel and kindred mercurials. Several elaborate articles have recently appeared, claiming for this agent all the remedial powers claimed for it by the fathers. Thus we progress in circles. However, one hobby is about as good as another, and "often a great deal better."

The famous Milwaukee surgical feat, extirpation of a kidney, has recently been accomplished by Simon, at Heidelberg. It seems a country surgeon had performed ovariotomy on the patient, and had injured the right ureter, giving rise to a urinary fistula. After several unsuccessful attempts to cure the fistula, Simon cut in from the lumbar region, shelled the kidney out of its capsule, tied *en masse*, and cut it away. The patient was cured. Simon now proposes extirpation of the kidney for such diseases as echinococcus, abscess, hydronephrosis, and renal calculi. T. Spencer Wells, in commenting on this case, recommends treatment of renal cysts by tapping and drainage, and has advised nephrotomy in some cases of renal calculi. The writer has at present under treatment an aggravated case of renal calculus, with frequent hæmaturia, colic and occasional pyelitis. What do our correspondents say as to our asking Professor Gunn to cut for it?

BROWN SEQUARD claims to have demonstrated in the spinal cord a special set of motor nerves, distinct from the voluntary motor, —the former being more in the lateral columns, and the latter more in the anterior. He thinks in epilepsy, these special fibres are the ones especially involved, and not the voluntary motor fibres. This is in harmony, he says, with Dr. Charet's statement, that inflammation (sclerosis) of the lateral columns of the spinal cord is invariably accompanied by muscular spasms.

A SEVERE influenza has been recently visiting London. "It usually begins quite suddenly with pain in the throat and ears, followed by shivering, *malaise*, constriction of the chest, sneezing and utter prostration. The temperature goes up to 102 degrees the first night, and there is some tendency to light-headedness. The *Medical Times and Gazette* says: "Bed, lemonade and beef tea, with an aperient pill and ammonia draught, the first day; next morning a five-grain dose of quinine, and half a pint of champagne at night, form the best way of dealing with this unpleasant visitor—*tuto*, *cito*, *et jucunde*."

DR. WHITE reports to the Boston Society for Medical Improvement several cases of Lupus successfully treated by Galvano-caustic. Dr. Hodges eulogized the same treatment, and also commends it in nævus and hæmorrhoids.

WE fear that our Boston friends, who discovered anæsthesia, do not mourn as they should over the death of the young lady to whom Sir James Y. Simpson recently administered chloroform.

DR. BARCLAY (*Dublin Press*) thinks that two or three drops of chloroform taken three or four times a day, is an admirable solvent for biliary calculi.

PROF. HOWE (*Eclectic Journal*,) reports successful treatment of *mania a potu* hypodermically. The remedies used were in these proportions: ℞. Water ℥ iv; morphia. gr. ij; atropia gr. j; M. S.—Inject, hypodermically, from thirty to sixty drops.

DR. FERRAND (*Edinburgh Journal*) urges the possibility of securing the effects of iodide of potassium applied in the dry form externally. In one case he secured iodism by putting on his patient a shirt previously dipped in a solution containing two drachms of the iodide, and then dried. After three days this shirt was replaced by another prepared in the same way; but on the fourth day the iodic symptoms occurred.

THE National Medical Society lately organized at Washington, "embraces all colors, sexes and sects in medicine," which he of the *Nashville Journal* ethnologically christens "Organic Mosaic."

RHIGOLENE is not explosive, but it should be kept in a cool place; or when it is wanted for local anæsthesia, it will be found to have evaporated.

THE *Detroit Journal* says that chloral is most conveniently administered in the form of a mixture containing not over forty grains to the fluid ounce, in simple syrup, flavored with peppermint. As chloral is getting cheaper, of course every body will try it. Confidence, in medicine at least, is not a plant of slow growth, as was demonstrated the other day by a physician who said that all that was needed to cure a certain patient was to pass a bougie every day and take chloral. A week after, "our Moses" took from that patient's bladder stones enough to start in the business of street paving.

PROF. WORMLEY, of Sterling Medical College, has isolated and described *gelsemia*, the true, active alkaloid of gelsemium.

DR. MCGRAW, of Detroit, recommends the use of Fl. Ext. Belladonna, in five-drop doses *ter die*, several days before expected labor. The belladonna, or stramonium suppositor is better. —— The *Medical Investigator* don't believe that *pulsatilla*; third trituration, will cause breech presentation, and consistently says: "The use of *pulsatilla* in mal-presentations, as a substitute for turning, we shall choose to regard worse in the *breach* than in the observance." *We* think so.

THE same journal reports Scarlatina still prevailing in Chicago. " *Calc. C.* is very frequently indicated. When the eruption is tardy in making its appearance, and cerebral symptoms are prominent, *Apis* has proved efficient. *Bell.*, strange to say, has not been often indicated." *We* are surprised, also, about *Bell.*

HAS *capsicum annuum* any real " Homœopathicity to hooping cough?"

THE " first attenuation " of Petroleum being recommended for sea-sickness, the *Medical Gazette* wants to know if it is on the principle that " *rock oil*" is an appropriate " similium " to still the rocking of the waves. Evidently the *Gazette* man " makes light " of the petroleum. " No levity."

PROF. CHISOLM (*Baltimore Medical Journal*) discards dressings after the incision for *fistula in ano.* Immediately after cutting, he applies, with a camel's-hair brush, or sponge, the liquid persulphate of iron over the entire surface. If much hæmorrhage occurs, he introduces, for a few hours, a dossil of lint soaked in the solution. Nothing more is needed save ablutions for cleanliness, and the patient is not often compelled to keep in bed, or in the house.——In the olden time, *Tulpius* and others, believed anal fistula " very high, either to the Loins, or the *Vertebræ* of the Breast, or sometimes to the Shoulders; whose inaccessible *Caries* the tortuous windings of the *fistula* does hinder from being searched with a probe, which also hinders injections, designed to cleanse the Ulcer, and does exclude the Hand, which might take out the vititated bone. Which, nevertheless, not being timely taken away, the Patient dies before his time, and the *fistula*, deriving its original from a remote *Caries*, does obstinately resist the Physician's cure. Whose lips though you clip open and ampliate,(which yet is very good in cutaneous fistula's,) nevertheless you will lose your labour, and you can never come to the farthest end of these sinuous windings, from whence so many branches, and so frequent rivulets descend by muscles and tendons, which lie deep, that though a Probe be never so dextrously put into such a tortuous *fistula*, yet it can never reach or remove the *Caries*, that is the cause of a continual *fistula*."

